# Genetic characteristics and prognosis of m6A RNA methylation regulator in acute myeloid leukemia

**DOI:** 10.1016/j.gendis.2025.101789

**Published:** 2025-08-05

**Authors:** Kaili Liao, Daixin Guo, Yujie Hu, Jiarong Wen, Yuhan Xu, Xinyi Bai, Jinting Cheng, Beining Zhang, Xiaozhong Wang

**Affiliations:** a Jiangxi Province Key Laboratory of Immunology and Inflammation, Jiangxi Provincial Clinical Research Center for Laboratory Medicine, Department of Clinical Laboratory, The Second Affiliated Hospital, Jiangxi Medical College, Nanchang University, Nanchang, Jiangxi 330031, China; b School of Public Health of Nanchang University, Nanchang, Jiangxi 330006, China; c The First Clinical Medical College of Nanchang University, Nanchang, Jiangxi 330006, China; d Queen Mary College of Nanchang University, Nanchang, Jiangxi 330006, China

Acute myeloid leukemia (AML) is a malignant disease of myeloid stem/progenitor cells, marked by the proliferation of immature myeloid cells in the bone marrow and blood, leading to anemia, bleeding, infection, fever, and organ infiltration.^1^ It accounts for 30% of pediatric leukemia, impacting molecular biology and chemotherapy, and most cases have a poor prognosis.^2^ While the exact etiology is unknown, it is related to regional factors, radiation, chemicals, alcoholism, smoking, and viral infections.^3^ Genetic mutations and biomarkers suggest a combination of genetics and environment.^4^ m6A is a common mRNA modification, but little is known about its role in AML. This study examines the genetic traits and prognosis of m6A regulators in AML, using TCGA-AML samples to explore the relationship between changes in m6A regulators and clinicopathology, thereby advancing our understanding of RNA epigenetics in AML.

We downloaded AML RNA-sequencing transcriptome data (RPKM data) and clinical survival data (including disease characteristics) from ICGC and TCGA (https://portal.gdc.cancer.gov/) along with gene annotation files from GENECODE (https://www.gencodegenes.org/). We selected samples with pathological characteristics and RNA-sequencing expression data, resulting in 151 acute myeloid cell-like leukemia samples. Because there is no stage data for AML, we divided the AML samples into eight categories according to their morphological characteristics (M0 Undifferentiated, M1, M2, M3, M4, M5, M6, M7) (Table S1). Clinical information is summarized in Table S3. Table S4 provides information on 13 m6A RNA methylation regulators. Among the eight types of samples, we constructed the expression profiles of these 13 m6A RNA methylation regulators (Fig. 1A). Genes are categorized according to their functions: writers (methyltransferases: METTL3, METTL14, WTAP, KIAA1429, RBM15, ZC3H13), which catalyze mRNA m6A methylation; readers (binding proteins: YTHDC1, YTHDC2, YTHDF1, YTHDF2, HNRNPC), which recognize RNA methylation modifications and participate in translation, degradation, and miRNA processing; and erasers (demethylases: FTO, ALKBH5), which demethylate m6A-modified bases. To analyze samples consistently across two subgroups, we performed consensus clustering using ConsensusClusterPlus (k = 2) based on the expression levels of the 13 regulatory factors in 151 patients, yielding two subgroups, RM1 and RM2 (high expression group and low expression group) (Fig. 1B). RM1 and RM2 consist of 91 and 60 samples, respectively (Table S2). In both subgroups, the expression levels of the 13 m6A RNA methylation regulators in RM1 were generally higher than those in RM2 (Fig. 1C). To comprehensively analyze the pathological characteristics of the two subgroups, we conducted a *t*-test to compare age differences between RM1 and RM2. The box plot indicates an age difference between the two subgroups (*p* = 0.08), although this difference is not significant (Fig. S1A). We employed the Chi–Square test to analyze differences among the whole subclasses of the two subgroups, revealing significant differences in their morphological characteristics (*p* = 0.00337) (Fig. S1B). Additionally, we used Cox regression to analyze survival differences between the two subgroups and generated a Kaplan–Meier survival curve. RM2 (blue) patients exhibited longer survival times than RM1 (red) patients (Fig. S1C).

**Figure 1:**
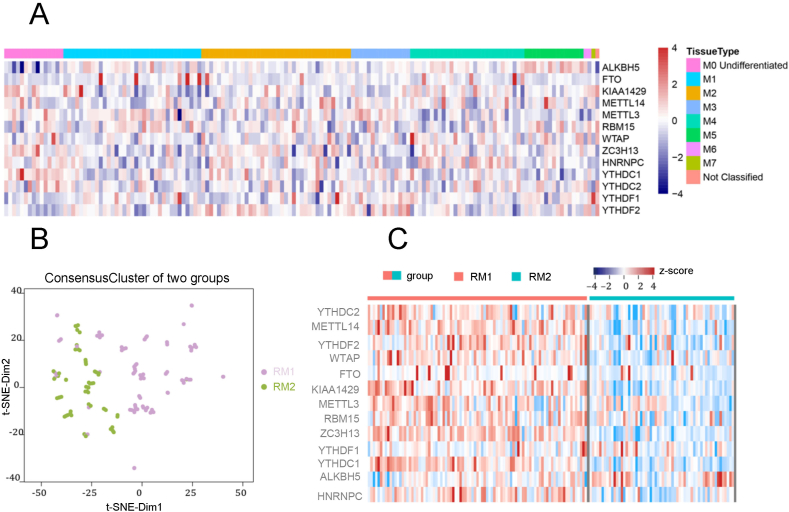
Insights from m6ARNA methylation regulators. **(A)** Heat map of m6A RNA methylation regulators among eight morphological features. **(B)** Consistent clustering of acute myeloid leukemia samples. **(C)** Heat map of 13 m6A RNA methylation regulators in two subgroups of acute myeloid leukemia.

To analyze the interaction between m6A RNA methylation regulators and their functions among subgroups, we utilized STRING to create a protein–protein interaction network diagram of the 13 m6A RNA methylation regulators (Fig. S1D). Subsequently, we performed principal component analysis to evaluate expression differences between the two subgroup samples (Fig. S2A) and utilized the R package clusterProfiler for enrichment analysis of the 13 m6A RNA methylation regulators (Gene Ontology (GO) biological processes and Kyoto Encyclopedia of Genes and Genomes (KEGG) pathway enrichment) (Fig. S2B). The 13 m6A RNA methylation regulators were mainly involved in the Notch signaling pathway, cytokine-mediated signaling pathway, endothelial pathways, and biological processes. Next, we conducted Cox regression analysis and risk scoring to predict prognosis. Each sample was scored based on the m6A RNA methylation regulators using the formula: Risk score = Σ (coefi ∗ xi), where “coefi” is the regression coefficient from the Cox regression and “xi” is the expression value of each prognostic methylation regulator in AML. The samples were divided into high- and low-risk groups based on their risk scores, and survival analysis was performed for each group. The Kaplan–Meier survival curve is shown in Figure S3A, demonstrating that the risk score effectively separates the high- and low-risk groups (*p* = 0.042). To further explore the relationships among the 13 m6A RNA methylation regulators, we constructed a co-expression relationship diagram to represent the functional correlations between different methylation regulators. This diagram was visualized using the R package Corrplot and is presented in Figure S3B. The *p*-value chart (Table S5) shows the correlation between different m6A RNA regulators, underscoring the importance of statistically validating these correlations to establish their significance and relevance.

To predict the prognosis and clinicopathological characteristics of tumor patients, we employed risk scores calculated by the features. Receiver operating characteristic curves were used to estimate classification performance, with a higher area under the curve indicating better performance. We used the 13 m6A RNA methylation regulators as combined features, mapped them to TCGA-AML, and employed Support Vector Machine (SVM) analysis to assess the risk score of combined features and predict three-year survival outcomes. The training set is shown in Figure S4A, and the test set is shown in Figure S4B. Risk scores for subgroup outcome prediction are displayed in Figure S4C for the training set and Figure S4D for the test set. Risk scores were also used to evaluate combined features for outcome prediction, with the training set shown in Figure S4E and the test set shown in Figure S4F. The risk score of the combined features was used to predict the morphological characteristics of the outcome, with the training set shown in Figure S4G and the test set shown in Figure S4H. The receiver operating characteristic curve demonstrates that the risk score accurately predicts the three-year survival rate of AML patients. In the RM1 and RM2 subgroups, the risk score outperformed morphological features in predicting prognosis and clinicopathological characteristics, confirming its effectiveness. To analyze the prognostic correlation of the 13 m6A RNA methylation regulators, we individually assessed each regulator for prognosis and drew survival curves (Fig. S5). The results indicated that the erasers (demethylases) FTO and ALKBH5 and the writer ZC3H13 were significantly correlated with overall survival (*p* < 0.05; Fig. S5K–M).

In conclusion, 13 major m6A RNA methylation regulators were classified into two subgroups (RM1 and RM2) through consistent clustering analysis. Functional analysis and STRING database-based protein–protein interaction network analysis revealed interactions among these regulators. Principal component analysis demonstrated significant expression differences between RM1 and RM2. Survival analysis suggested that FTO, ALKBH5, and ZC3H13 are key regulatory genes, with AML leading to reduced survival time. We downloaded data from external datasets GSE47051 and GSE10255 to validate the model. Using the ConsensusClusterPlus package, we divided the data into two clusters (Fig. S6, 7). We confirmed through heatmap analysis that there were significant gene expression differences between the two clusters, demonstrating the effectiveness of the model (Fig. S8, 9). Compared with RM2, RM1 has a worse prognosis and more morphological features. Moreover, gene enrichment analysis revealed that functional processes, such as endothelial-hematopoietic transformation transmitting signals through the Notch pathway, were significantly enriched in RM1. These findings indicate that the 13 m6A RNA methylation enzyme regulators can be used as a risk profile for AML, serving as both an independent prognostic marker and a predictor of the clinicopathological features of AML.

## CRediT authorship contribution statement

**Kaili Liao:** Conceptualization, Formal analysis, Investigation, Writing - original draft, Visualization, Writing - review & editing. **Daixin Guo:** Formal analysis, Investigation, Writing - original draft, Writing - review & editing. **Yujie Hu:** Formal analysis, Investigation, Writing - original draft, Writing - review & editing. **Jiarong Wen:** Investigation, Data curation, Writing - review & editing. **Yuhan Xu**: Writing - original draft. **Xinyi Bai:** Investigation. **Jinting Cheng:** Investigation. **Beining Zhang:** Investigation. **Xiaozhong Wang:** Project administration, Methodology, Supervision, Writing - review & editing, Funding acquisition.

## Data availability

The microarray data were obtained from the GTEx, TCGA, TIMER, CCLE, GSEA, and KEGG databases.

## Funding

This work was supported by National Natural Science Foundation of China Project (No. 82460036), Jiangxi Province Key Laboratory of Immunology and Inflammation, Jiangxi Provincial Clinical Research Center for Laboratory Medicine, Department of Clinical Laboratory, The Second Affiliated Hospital, Jiangxi Medical College, Nanchang University,Nanchang, Jiangxi, China, and the Science and Technology Plan Project of Jiangxi Provincial Department of Science and Technology (No. 20212ACB206016).

## Conflict of interests

The authors declared that the research was conducted in the absence of any commercial or financial relationships that could be construed as a potential conflict of interests.

## Figures and Tables

**Figure S1:**
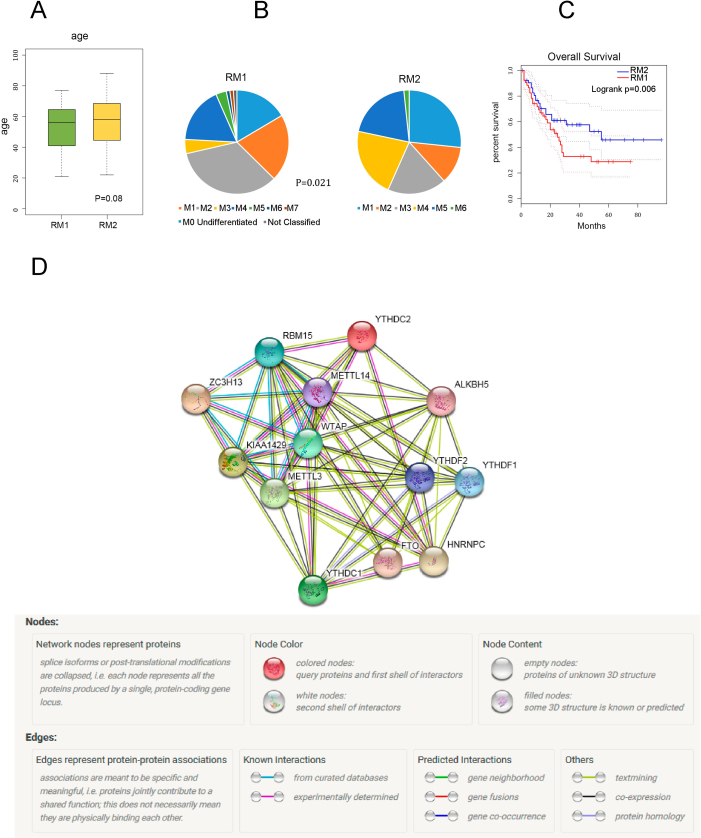
Analyzing subgroups of acute myeloid leukemia. **(A)** Age box between two subgroups of acute myeloid leukemia. **(B)** Pie chart of morphological characteristics between the two subgroups of acute myeloid leukemia. **(C)** Survival curve between two subgroups of acute myeloid leukemia. **(D)** Protein interaction network (PPI) of 13 m6A RNA methylation regulators.

**Figure S2:**
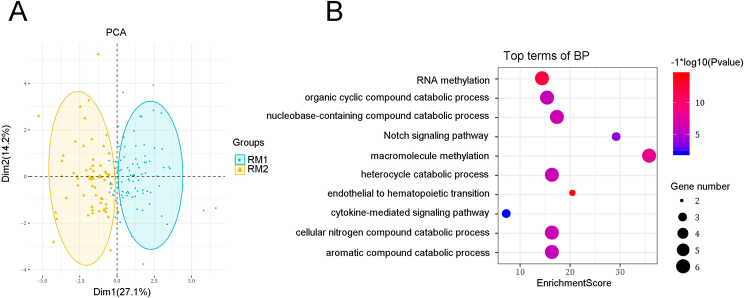
Subgroup analysis of principal components and m6A RNA methylation regulators. **(A**) Principal component analysis of two subgroups (RM1, RM2). **(B**) Enrichment map of m6A RNA methylation regulators in two subgroups (RM1, RM2).

**Figure S3:**
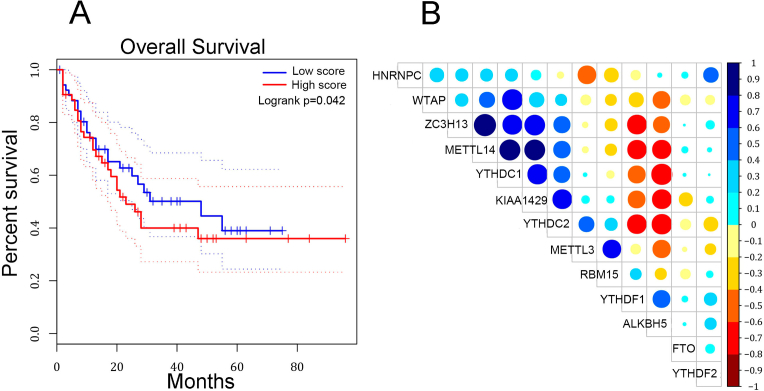
Subgroup analysis of principal components and m6A RNA methylation regulators. **(A)** Sample survival curve for high- and low-risk groups. **(B**) Correlation diagram of methylation regulator expression in acute myeloid leukemia. The beginning of each line represents 13 m6A RNA methylation regulators, and each small circle represents the Spearman correlation coefficient of two methylation regulators. The red indicates that the Spearman correlation coefficient is close to −1, and the blue indicates that the Spearman correlation coefficient is closer to 1. The closer the absolute value of the Spearman correlation coefficient is to 1, the more speculated that the two methylation regulators play a similar role in RM1 or RM2 of acute myeloid leukemia.

**Figure S4:**
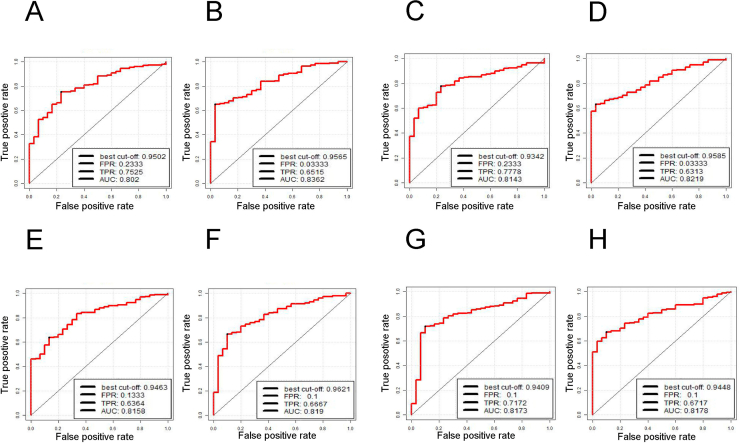
Predictive analysis of combined feature risk scores. **(A**) Prediction effect of risk scores of combined features on three-year survival results (training set). **(B)** Prediction effect of risk scores of combined features on three-year survival results (test set). **(C)** Prediction effect of risk scores of combined features on subgroups (training set). **(D)** Prediction effect of risk scores of combined features on subgroups (test set). **(E)** Prediction effect of risk scores of combined features on prognosis results (training set). **(F)** Prediction effect of risk scores of combined features on the prognostic results (test set). **(G)** Prediction effect of risk scores of combined features on the morphological features (training set). **(H)** Prediction effect of risk scores of combined features on the morphological features (test set).

**Figure S5:**
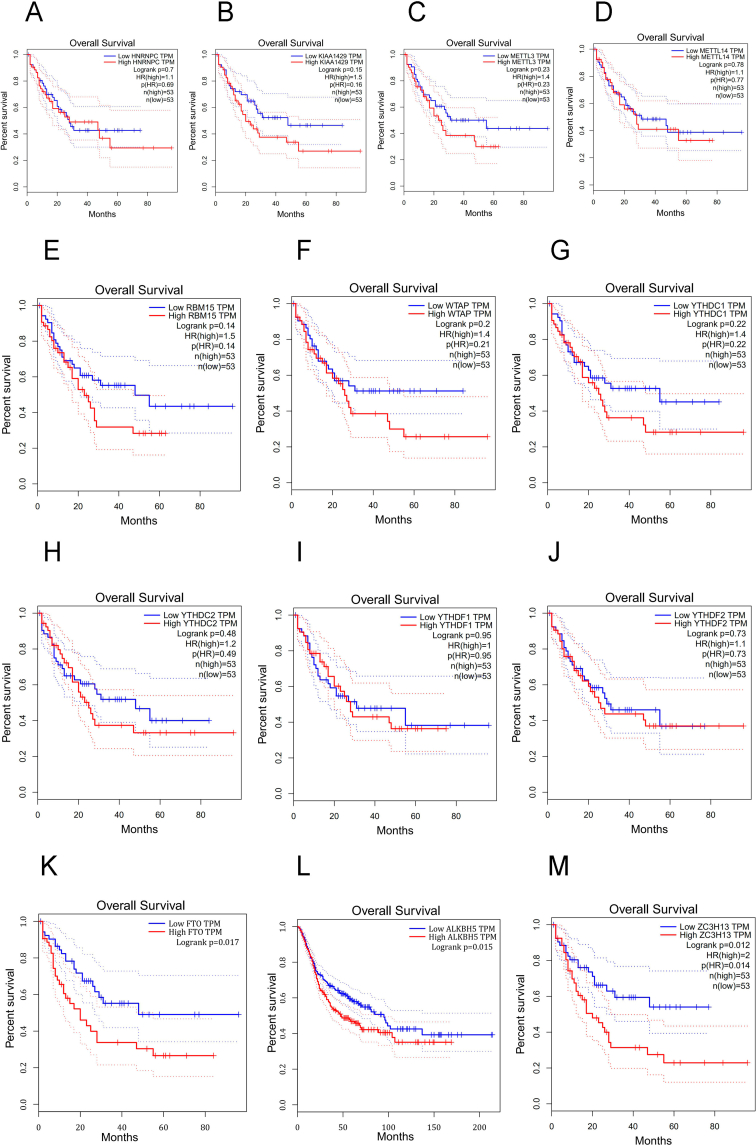
Prognostic analysis of 13 m6A RNA methylation regulators.

**Figure S6:**
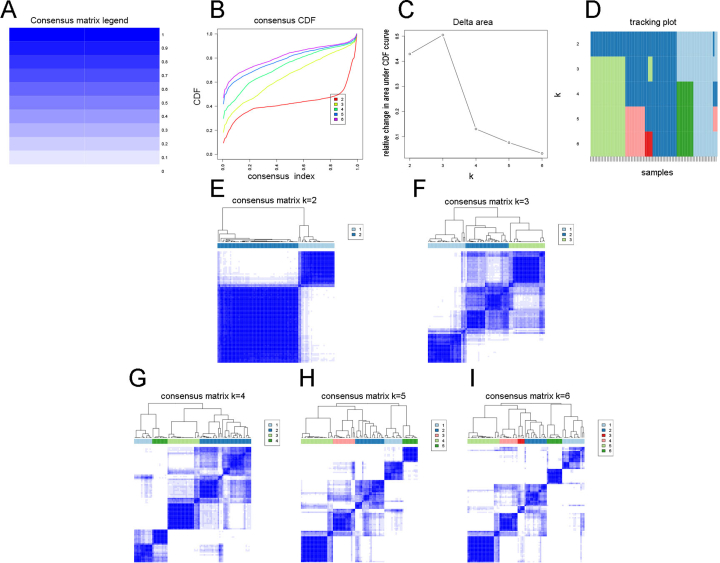
Consensus cluster analysis of external dataset GSE47051.

**Figure S7:**
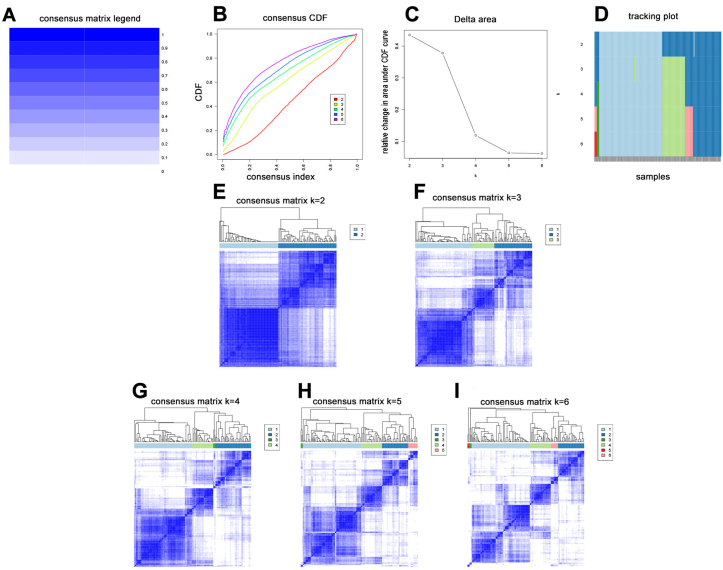
Consensus cluster analysis of external dataset GSE10255.

**Figure S8:**
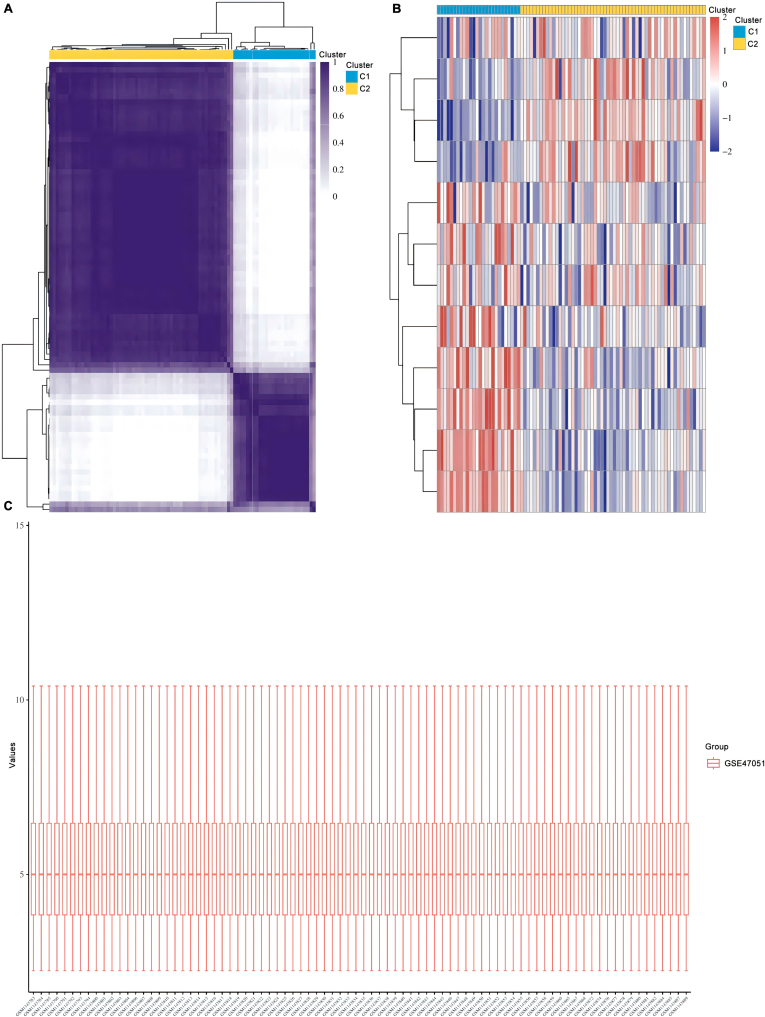
Heatmap of the differences in the expression of prognostic lncRNAs between two clusters of GSE47051.

**Figure S9:**
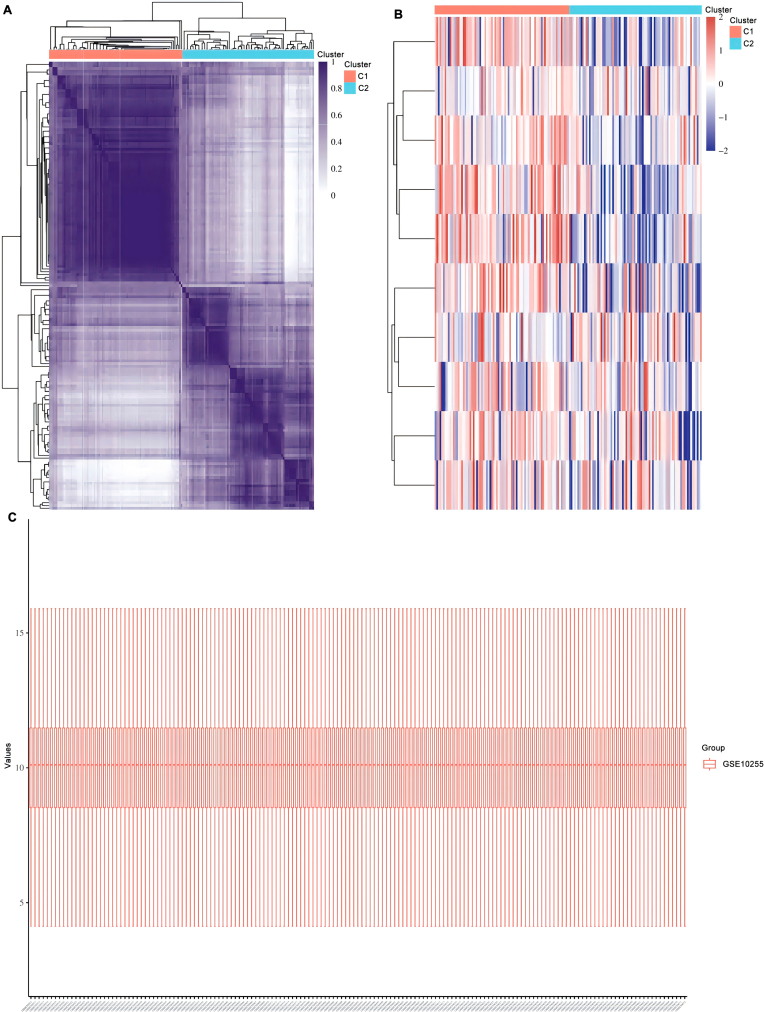
Heatmap of the differences in the expression of prognostic lncRNAs between two clusters of GSE10255.
