# Supplementary material for: Genetic characteristics and prognosis of m6A RNA methylation regulator in acute myeloid leukemia
Source: Genes Dis. 2025 Aug 5;13(1):101789. doi: 10.1016/j.gendis.2025.101789 (PMC12624708; doi:10.1016/j.gendis.2025.101789)
Supplement: Multimedia component 1 [file mmc1.docx]

**2 Materials and methods**

2.1 Data acquisition and preprocessing

Download acute myeloid leukemia (AML) RNA-seq transcriptome data (rpkm data), acute myeloid leukemia clinical survival data (including disease) from ICGC and TCGA (https://portal.gdc.cancer.gov/) (Physical characteristics and survival time, etc.), download gene annotation files from GENECODE (https://www.gencodegenes.org/). Select samples that have both pathological characteristics and RNA-seq expression data, and a total of 151 acute myeloids cell-like leukemia samples were obtained. Since there is no stage data for AML, leukemia morphology: M0 Undifferentiated, M1, M2, M3, M4, M5, M6, M7 were used as a pathological feature. From the literature [8-10], 13 widely reported m6A RNA methylation regulators (m6A RNA methylation regulators for different participating roles in the methylation process: writers (methyltransferase) -METTL3, METTL14, WTAP, KIAA1429, RBM15, ZC3H13; readers (binding proteins)-YTHDC1, YTHDC2, YTHDF1, YTHDF2, HNRNPC; erasers (demethylase)-FTO, ALKBH5). The selection process for m6A RNA methylation regulators is: We first collated a list of sixteen m6A RNA methylation regulators from published literature, [1-3] and then we restricted the list to genes with available RNA expression data in the TCGA datasets. This yielded a total of thirteen m6A RNA methylation regulators. Then, we systematically compared the expression of these m6A RNA methylation regulators with different clinical pathological features.

Select 151 acute myeloid leukemia samples with existing pathological characteristics (morphological characteristics: M0 Undifferentiated, M1, M2, M3, M4, M5, M6, M7) and RNA-seq expression data; use gene annotation file to construct the expression profile (rpkm data) of m6A RNA methylation regulators (**Supplementary Table 1**), classify acute myeloid leukemia samples based on the morphological characteristics of acute myeloid leukemia, and use m6A RNA methylation regulators, finally construct an expression heat map.

2.2 Sample consistent clustering and subgroup analysis

The AML m6A RNA methylation regulator was used as a feature vector, and ConsensusClusterPlus consensus clustering (k = 2) was performed on the samples. Two subgroups RM1 and RM2 were obtained. The t-test was used to compare and analyze the age difference between rm1 and rm2, the chi-square test was used to analyze the difference between the who subclasses of the two subgroups, and the cox regression was used to analyze the difference in survival between the two subgroups.

2.3 Analysis of the interaction between m6A RNA methylation regulators and functional analysis between subgroups

The STRING database was used to analyze the interactions between m6A RNA methylation regulators, and Spearman analyzed the expression correlation of m6A RNA methylation regulators. Construct the expression profiles of m6A RNA methylation regulators in RM1 and RM2 subgroups, use PCA to analyze the differences in m6A RNA methylation regulator expression between the two subgroups, and use R package: clusterprofier to annotate and Enrichment analysis. The enrichment content includes GO biological processes (BP) and KEGG Pathways.

2.4. Cox regression analysis and the use of risk scores to predict prognosis and pathological characteristics

A risk score is given for each acute myeloid leukemia sample, with the formula:

Risk score = $\sum_{i=1}^{n} Coefi*xi$, Among them, Coefi is the regression coefficient (COefficient) of COX regression, and xi is the expression value of the prognosis methylation regulator of each acute myeloid leukemia. This formula was used to calculate the risk score of each acute myeloid leukemia sample. According to this risk score, the sample was divided into high-risk group and low-risk group. Find the difference between overall survival (OS) between the two categories.

2.5. Prediction of prognosis and clinicopathological characteristics of tumor patients using risk scores calculated by features

Receiver operating characteristic (ROC) curves were used to estimate classification performance.The higher the area under the curve (AUC) value, the higher the classification performance. Using 13 m6A RNA methylation regulators as risk characteristics, the TCGA acute myeloid leukemia sample was divided into 5 parts, 5 times cross-validation was applied, the model was trained with four fifths of the sample and tested on the test set (the remaining one fifths of the samples). In this way, each part will be tested once. Then the receiver operating characteristic (ROC) curve is used to estimate the classification performance. The higher the area under the curve (AUC) value , The higher the classification performance. Comparative analysis of whether the risk score model can perfectly predict the three-year survival rate, RM1 / 2 subgroup, prognosis results, morphological characteristics and other characteristics of tumor patients.
