# Supplementary material for: Genetic characteristics and prognosis of m6A RNA methylation regulator in acute myeloid leukemia
Source: Genes Dis. 2025 Aug 5;13(1):101789. doi: 10.1016/j.gendis.2025.101789 (PMC12624708; doi:10.1016/j.gendis.2025.101789)
Supplement: Multimedia component 4 [file mmc4.doc]

**Tables**

**Table S3 Clinical factors**

| Clinical factors | |  | |  |
| --- | --- | --- | --- | --- |
| leukemia_morphology | |  | |  |
|  | | M0 Undifferentiated | | 15 |
|  | | M1 | | 35 |
|  | | M2 | | 38 |
|  | | M3 | | 15 |
|  | | M4 | | 29 |
|  | | M5 | | 15 |
|  | | M6 | | 2 |
|  | | M7 | | 1 |
|  | | Not Classified | | 1 |
| Vital_status | |  | |  |
|  | | alive | | 54 |
|  | | dead | | 97 |
| Age | |  | |  |
| <30 | 13 | |  | |
|  | | 30-40 | | 18 |
|  | | 41-50 | | 23 |
|  | | 51-60 | | 30 |
|  | | 61-70 | | 38 |
|  | | 71-80 | | 26 |
|  | | 81-90 | | 3 |

**Table S4 Information of m6A RNA regulators**

| m^6^A RNA regulators | Chr | Start | End | Strand | Type |
| --- | --- | --- | --- | --- | --- |
| ALKBH5 | 17 | 18183078 | 18209954 | + | erasers |
| FTO | 16 | 53701692 | 54158512 | + | erasers |
| KIAA1429 | 8 | 94487693 | 94553529 | - | writers |
| METTL14 | 4 | 118685368 | 118715433 | + | writers |
| METTL3 | 14 | 21498133 | 21511375 | - | writers |
| RBM15 | 1 | 110338506 | 110346681 | + | writers |
| WTAP | 6 | 159725585 | 159756319 | + | writers |
| ZC3H13 | 13 | 45954465 | 46052759 | - | writers |
| HNRNPC | 14 | 21209136 | 21269494 | - | readers |
| YTHDC1 | 4 | 68310387 | 68350089 | - | readers |
| YTHDC2 | 5 | 113513683 | 113595285 | + | readers |
| YTHDF1 | 20 | 63195429 | 63216234 | - | readers |
| YTHDF2 | 1 | 28736621 | 28769775 | + | readers |

**Table S5 Correlation Significance of m6A RNA Methylation Regulators**

| m6A RNA regulators | ALKBH5 | FTO | KIAA1429 | METTL14 | METTL3 | RBM15 | WTAP | ZC3H13 | HNRNPC | YTHDC1 | YTHDC2 | YTHDF1 | YTHDF2 |
| --- | --- | --- | --- | --- | --- | --- | --- | --- | --- | --- | --- | --- | --- |
| ALKBH5 |  | 0.94 | 0.01 | 0.02 | 0.04 | 0.43 | 0.06 | 0.09 | 0.89 | 0.00 | 0.01 | 0.06 | 0.44 |
| FTO | 0.94 |  | 0.27 | 0.81 | 0.90 | 0.55 | 0.53 | 0.97 | 0.79 | 0.96 | 0.51 | 0.83 | 0.59 |
| KIAA1429 | 0.01 | 0.27 |  | 0.00 | 0.69 | 0.71 | 0.20 | 0.00 | 0.54 | 0.01 | 0.01 | 0.05 | 0.76 |
| METTL14 | 0.02 | 0.81 | 0.00 |  | 0.83 | 0.41 | 0.15 | 0.00 | 0.38 | 0.00 | 0.07 | 0.02 | 0.91 |
| METTL3 | 0.04 | 0.90 | 0.69 | 0.83 |  | 0.03 | 0.54 | 0.85 | 0.03 | 0.91 | 0.16 | 0.56 | 0.47 |
| RBM15 | 0.43 | 0.55 | 0.71 | 0.41 | 0.03 |  | 0.45 | 0.35 | 0.23 | 0.73 | 0.35 | 0.47 | 0.77 |
| WTAP | 0.06 | 0.53 | 0.20 | 0.15 | 0.54 | 0.45 |  | 0.34 | 0.24 | 0.00 | 0.45 | 0.21 | 0.60 |
| ZC3H13 | 0.09 | 0.97 | 0.00 | 0.00 | 0.85 | 0.35 | 0.34 |  | 0.35 | 0.01 | 0.09 | 0.01 | 0.64 |
| HNRNPC | 0.89 | 0.79 | 0.54 | 0.38 | 0.03 | 0.23 | 0.24 | 0.35 |  | 0.38 | 0.79 | 0.59 | 0.17 |
| YTHDC1 | 0.00 | 0.96 | 0.01 | 0.00 | 0.91 | 0.73 | 0.00 | 0.01 | 0.38 |  | 0.10 | 0.05 | 1.00 |
| YTHDC2 | 0.01 | 0.51 | 0.01 | 0.07 | 0.16 | 0.35 | 0.45 | 0.09 | 0.79 | 0.10 |  | 0.01 | 0.22 |
| YTHDF1 | 0.06 | 0.83 | 0.05 | 0.02 | 0.56 | 0.47 | 0.21 | 0.01 | 0.59 | 0.05 | 0.01 |  | 0.34 |
| YTHDF2 | 0.44 | 0.59 | 0.76 | 0.91 | 0.47 | 0.77 | 0.60 | 0.64 | 0.17 | 1.00 | 0.22 | 0.34 |  |
